# Familial prevalence of psoriasis in patients with multiple sclerosis – results from an Australian survey

**DOI:** 10.3389/fneur.2025.1663031

**Published:** 2025-11-06

**Authors:** Varitsara Mangkorntongsakul, Ariadna Fontes-Villalba, Chun-Ting Justin Kwong, Olivia Charlton, Helen Marie McGuire, Turlough Montague, Kevin Phan, Venkatesha Venkatesha, Geoffrey Herkes, Saxon Smith, John Parratt

**Affiliations:** 1 Faculty of Medicine and Health, Northern Clinical School, The University of Sydney, Darlington, NSW, Australia; 2 Department of Neurology, Royal North Shore Hospital, St Leonards, NSW, Australia; 3 Faculty of Medicine and Health, School of Medical Sciences, The University of Sydney, Darlington, NSW, Australia; 4 Department of Dermatology, Royal North Shore Hospital, St Leonards, NSW, Australia; 5 Department of Dermatology, Liverpool Hospital, NSW, Australia; 6 Northern Sydney Local Health District Executive, Royal North Shore Hospita, St. Leonards, NSW, Australia; 7 Department of Dermatology, Sydney Adventist Hospital, Wahroonga, NSW, Australia; 8 The Dermatology and Skin Cancer Centre, Gosford, NSW, Australia; 9 ANU Medical School, ANU College Health and Medicine, The Australian National University, Canberra, ACT, Australia

**Keywords:** psoriasis, multiple sclerois, neuroimmunology, autoimmune disease, Australian population, familial study, survey study

## Abstract

**Background:**

The familial association of psoriasis with multiple sclerosis (MS) has been observed in several international studies. Whether this link is applicable in an Australian setting has yet to be established. The aim of this study was to evaluate the prevalence of psoriasis in family members of an MS cohort in the Australian population.

**Methods:**

A survey study of adult MS patients was conducted between 2018 and 2021 at the MS clinic, Royal North Shore Hospital, Sydney, Australia.

**Results:**

A total of 204 MS participants, with mean age of 48.8 years, were included in the study. Of these participants, 67 were male (32.8%) and 137 were female (67.2%). An increased rate of psoriasis was found among family members of MS patients: 35 (17.2%) MS patients had family history of psoriasis, and 11 patients reported a history of psoriasis themselves. Of the 35 MS patients with family history of psoriasis, female participants were found to be at a higher risk of psoriasis (34.6%, *n* = 9/26) as compared to their male counterparts (22.2%, *n* = 2/9).

**Discussion:**

This study demonstrates a significantly increased risk of psoriasis among family members of MS patients, suggesting overlap in genetic risk between the two immune-mediated diseases. Further prospective research is warranted to determine the characteristics of association in MS individuals with psoriasis or/and family history of psoriasis.

## Introduction

Psoriasis and multiple sclerosis (MS) are genetically multifaceted, chronic inflammatory conditions characterized by dysregulation of the adaptive immune system (1–3). The aetiologies of these disorders are complex interplays between genetic and environmental factors, as suggested by their wide phenotypic heterogeneity (1, 2). Importantly however, both disorders are primarily T-cell mediated, with T helper 1 (Th1) and Th17 pathways implicated in pathogenesis. Moreover, interleukin (IL)-23 and tumour necrosis factor alpha (TNFα) have been demonstrated to be key in the development of plaques in both diseases (4, 5). Several studies have determined multiple common susceptibility genes and pathogenic mechanisms are shared between psoriasis and MS. (6–9) Clustering of psoriasis within family members of MS patients may reflect common immunogenetic mechanisms, interacting with disease specific triggers. Whilst it is well established that MS is polygenically inherited, specific human leukocyte antigen (HLA) class II alleles, including HLA-DRB1*15:01, HLA-DRB1*13:03, HLA-DRB1*03:01, HLA-DRB1*08:01 and HLA-DQB1*03:02 are associated with increased risk, particularly amongst MS patients and their relatives (5, 10). Despite several shared pathways of immune dysregulation in both disease settings, not all subtypes of MS are linked to psoriasis (4, 5). Other key differences in underlying pathology are exhibited by TNF inhibitors demonstrating benefit in the setting of psoriasis and psoriatic arthritis but exacerbating demyelinating events in MS. (5) Indeed, Tumour necrosis factor receptor (TNFR) 1 and TNFR 2 represent promising therapeutic targets in disease modifying therapy of MS. TNFR 1 mediates demyelination and apoptosis whereas TNFR2 promotes remyelination and immune modulation (11–13). Therapeutic effects of Atrosab, a selective TNFR1 blocker, has demonstrated a reduction in clinical severity in an animal model of autoimmune encephalomyelitis (11). However, its effects in humans appear paradoxical, limiting the translation from animal models to clinical practice (14).

Though the global prevalence of psoriasis (2–3%) and MS (0.3%) vary across geographical regions and ethnic backgrounds, those of Caucasian with European origins and in high-income nations exhibit higher prevalence of both autoimmune diseases (3, 15–17). The increased concordance of psoriasis in MS patients was first noted in 1989 by Cendroswki et al. (9). Since then, the incidence of this association has been steadily rising, with the risk remaining high despite an adjustment made for a family history of psoriasis (6–8). The epidemiological and familial association between psoriasis and MS is well established in the global setting. Indeed, several studies showed a strong familial link between psoriasis and MS, reporting a higher prevalence of psoriasis among relatives of MS patients (18–23). This observation was further supported by a recent systematic review and meta-analysis performed by Charlton et al., suggesting a greater risk of developing psoriasis in first-degree family members of patients with MS. (5)

While there is considerable data documenting a potential familial association between psoriasis and MS, this link has not been previously explored within an Australian cohort. The primary aim of this study was to investigate the familial frequency of psoriasis and MS among the adult Australian population.

## Materials and methods

This single centre study was conducted from July 2018 to February 2022, at a large MS outpatient clinic, Royal North Shore Hospital (RNSH), Sydney, Australia.

A survey consisting of 29 questions (Appendix 1) was distributed to patients, aged 18 years or above, with a confirmed diagnosis of MS according the 2017 McDonald criteria (24), see Mangkorntongsakul et al. (under parallel review in Frontiers in Neurology (submission ID 1663015), whereas a diagnosis of psoriasis was based on self-report by the participants. The diagnosis was given either by their family physician or a dermatologist. Interested subjects were randomly invited to participate in this survey either via e-mail or during their appointment at the MS outpatient clinic. Data was collected and stored through the REDCap platform. The questionnaires were designed to capture the prevalence and incidence of familial psoriasis, and to ascertain the signs and symptoms of inflammatory dermatoses, indicative of psoriasis. We defined familial as the presence of any blood relative, regardless of degree of separation, with a self-reported diagnosis of psoriasis. The study was approved by the Northern Sydney Local Health District Human Research Ethics Committee, which is constituted in accordance with the National Statement on Ethical Conduct in Human Research, 2007 (NHMRC)(2021/ETH00892).

The data were presented as mean, standard deviation (symmetric normal data), median, range (skewed data or ordinal data), and proportions, with a 95% confidence interval (CI). Chi-square test or Fisher’s exact test were used to assess the association between two categorical variables. Binary logistic regression was used to estimate and test the significance of the odds of skin manifestation in MS patients. All the statistical tests were performed at a 0.05 level of significance. All statistical analyses were performed in SPSS V26.0.

## Results

A total number of 204 MS subjects, with a mean age of 48.8 years (range: 17–81) completed the survey (Table 1). Of these participants, 67 were male (32.8%) and 137 were females (67.2%). The percentage of patients diagnosed with psoriasis was 13.73% (*n* = 28/204). There was no significant difference in prevalence of psoriasis between males and females (9.0%, *n* = 6/67 for males and 16.1%, 22/137 for females, *p* = 0.166).

**Table 1:** Demographics of Multiple Sclerosis (MS) patient with and without a diagnosis of psoriasis.

	MS with psoriasis (*n* = 28)	MS without psoriasis (*n* = 176)	*p*-values
Gender			
Female	22 (78.6%)	115 (65.3%)	0.166
Male	6 (21.4%)	61 (34.7%)	
Age, mean (SD)	50.8 (13.7)	48.4 (13.6)	0.405

The overall prevalence of familial psoriasis among MS patients was 17.2% (35/204), 95% CI: 12.56–23.00% (Table 2). Among the 35 MS patients with familial psoriasis, 11 (31.4%) reported a personal history of psoriasis. Conversely, twenty-four MS patients with familial psoriasis had no personal history of psoriasis. Females were more likely than males to have psoriasis if there was a family history (females 34.6% *n* = 9/26, males 22.2%, *n* = 2/9), however the odds ratio was not significantly different between genders (OR = 1.85, 95% CI: 0.32–10.85, *p* = 0.494).

**Table 2:** Demographics of familial psoriasis in MS patients.

	Overall (*n* = 204)	MS patients			Familial psoriasis *n* = 35		
		Non-familial psoriasis (*n* = 169)	Familial psoriasis (*n* = 35)	p-values	With self-psoriasis diagnosis (*n* = 11)	Without self-psoriasis diagnosis (*n* = 24)	*p*-values
Gender							
Female, *n* (%)	137 (67.2)	111 (65.7%)	26 (74.3%)	0.324	9 (81.8)	17 (70.8)	0.685
Male, *n* (%)	67 (32.8)	58 (34.3%)	9 (25.7%)		2 (18.2)	7 (29.2)	
Age, mean (SD)	48.8 (13.6)	48.4 (13.6)	50.4 (14.0)	0.455	52.7 (10.9)	49.3 (15.3)	0.454

SD = standard deviation.

The chi-squared analysis indicated a statistically significant association between familial psoriasis and the diagnosis of psoriasis in MS patients, with a chi-squared value of 11.811 [1 degree of freedom] and a two-tailed *p*-value of 0.001 (Table 3). The corresponding binary logistic regression analysis indicated that the odds of self psoriasis diagnosis is 4.1 times for those with familial psoriasis as compared with those without familial psoriasis (OR = 4.1, 95% CI: 1.71–9.8, *p* = 0.001).

**Table 3:** Cross tabulation of familial psoriasis and diagnosis of psoriasis themselves.

Familial psoriasis	Psoriasis		Total
	No	Yes	
No	152 (89.9)	17 (10.1)	169 (100)
Yes	24 (68.6)	11 (31.4)	35 (100)
Total	176 (86.3)	28 (13.4)	204 (100)

Values within the parenthesis are percentages to the respective row totals.

Table 4 illustrates the results of binary logistic regression associated with odds of skin manifestations of various types for patients without a diagnosis of psoriasis compared to those with a diagnosis of psoriasis for the familial psoriasis subgroup in this survey study. The odds of thickened scaly skin of extensors behind ears or scalp and flaky, peeling, or scaly skin was 92% lesser for patients without the diagnosis of psoriasis as compared to those with a diagnosis of psoriasis (OR = 0.08, 95% CI: 0.01–0.41, *p* = 0.003). Similarly, odds of minimum six history of intermittent pruritus or erythematous rash and flaky peeling or scaly skin was significantly lesser for patients without the diagnosis of psoriasis as compared to those with a diagnosis of psoriasis in familial psoriasis group (OR = 0.07, 95% CI: 0.01–0.65, *p* = 0.019 and OR = 0.10, 95% CI:0.02–0.52, *p* = 0.006 respectively).

**Table 4:** Skin manifestations in MS patients with a family history of psoriasis relative to own diagnosis of psoriasis.

Variable	Skin manifestations		
	Odd Ratio	95% Cl	*p*-value
New skin manifestations since a diagnosis of MS	0.72	0.17–3.06	0.656
Minimum 6-month-history of intermittent pruritus or erythematous rash	0.07	0.01–0.65	0.019
Seasonal skin changes	0.60	0.14–2.58	0.493
Thickened scaly skin of extensors, behind ears or scalp	0.08	0.01–0.41	0.003
Flaky, peeling or scaly of skin	0.10	0.02–0.52	0.006
Dandruff	0.73	0.16–3.27	0.671
Nail changes	0.28	0.06–1.25	0.095

## Discussion

This single-centre family study demonstrated a high risk of familial psoriasis among Australian patients with MS (35 patients (17.2%), 95% CI: 12.56–23.00%). Although the observed prevalence was higher in females compared to males (OR = 1.85, 95% CI: 0.32–10.85, *p* = 0.494), this difference did not reach statistical significance at the *α* = 0.05 level. Nevertheless, existing literature has reported a female-to-male ratio of approximately 3:1 in MS (2), which aligns with our findings. This observed gender disparity in addition to the result of chi-squared analysis (χ^2^ = 11.811, *p* = 0.0008) revealed a statistically significant association between being diagnosed with psoriasis and having a family history of psoriasis, underscores the need to account for sex-specific factors when designing treatment strategies for MS patients, particularly those with a family history of psoriasis. Notably, up to 31.4% of MS participants with psoriasis have family history of psoriasis, compared to 10.1% of those without. This threefold increase was further supported by the logistic regression analysis, which demonstrated that MS patients with a family history of psoriasis were more than four times as likely to report a personal history of psoriasis compared to those without (OR = 4.1, 95% CI: 1.71–9.8, *p* = 0.001). Hence, these findings strongly suggest that genetic predisposition plays a key role in psoriasis risk among MS patients.

Furthermore, the study revealed that 9 out of 24 (38.0%) MS patients with a family history of psoriasis, but without a formal diagnosis of psoriasis, reported new skin manifestations over time. This implies that the incidence of skin diseases like psoriasis might be underdiagnosed in individuals with MS. Alternatively, this could relate to complications of MS disease modifying therapies, some of which are recognised to cause cutaneous adverse events including psoriasis (25).

To the best of our knowledge, this is the first Australian study which offers an insight to the link between MS and familial psoriasis. Several countries investigating a correlation of psoriasis in the families of definite MS patients established conflicting outcomes (5, 7, 18–21, 26). In 1996, Midgard et al., conducted a case–control study of 155 MS cases and 200 controls performed in Norway which intended to explore the relationship between MS and autoimmune disorders (27). Compared to the control group, a statistically significant increased risk of psoriasis was noted in MS patients with the mean age of onset ranging from 16 to 40 years old (OR = 2.96; 95% CI 1.23–7.66). However, no association was observed between familial prevalence of psoriasis and MS in this cohort (27).

On the contrary, UK and Greek studies suggested a stronger familial link. Broadley et al., demonstrated in the UK based case–control study (375 cases and 571 controls) that the prevalence of psoriasis was higher in relatives of MS multiplex families (families where multiple members are affected by the disease) compared with simplex families (families where only a single member has the disease), suggesting that genetic loading may partly explain the discrepancy (20). However, the overall risk of psoriasis observed in relatives of MS patients in this study was rather small, implying a limited contribution to MS genetic susceptibility (20). The Northern Greece large prospective case control study reinforced this finding. The study investigated the longitudinal prevalence of autoimmune disease among naïve MS patients for up to 7 years (21). The study consisted of 891 MS families of which 73 were multiplex families (292 members) and 818 were simplex families (2,820 members) versus 355 control families (1,580 members) (21). At baseline, a higher prevalence of psoriasis was detected among MS multiplex families (2.8%, *p* = 0.001), whereas no significant difference was noted within simplex families with MS (0.8%, *p* = 0.33). Nevertheless, in a comparison analysis between two points in time, a longitudinal increased frequency of psoriasis was revealed in both multiplex (3% *p* = 0.0002) and simplex (1.5%, *p* = 0.008) families of MS individuals.

It is noteworthy that these discrepancies between the Norwegian versus the UK/Greek studies may be explained by several methodological differences. The Norwegian cohort was a smaller case–control study, whereas the UK and Greek studies included larger sample sizes, prospective designs and family-matched controls. Moreover, the studies from the UK and Greek also explicitly analysed multiplex versus simplex families, better capturing the effect of genetic loading. Ethnic variation may have also contributed to the divergent findings.

Furthermore, Barcellos et al., conducted a large, well-characterised US family-based study of 176 families aiming at determining the prevalence of concurrent autoimmune disorders among MS patients and their first-degree relatives (19). Only families with the presence of two or more members with definite MS were recruited in this study. In an overall prevalence among first degree relatives of MS index cases (*n* = 1,317), psoriasis was observed to be the second most common autoimmune disease (2%) estimated in family members of MS, with the majority of the cases being male (19). This pattern was further supported by an Italian case–control study performed by Annunziata et al., exploring the link of psoriasis in MS families (22). In their study of 177 MS patients, psoriasis was denoted to be the most common autoimmune disease, affecting 30 relatives (16.9%) of MS participants. The prevalence of psoriasis in this cohort favoured a patriline; among 30 relatives with psoriasis, 16 of them were fathers of the subjects (*p* < 0.0001) (22). Additionally, a significant earlier onset of MS was demonstrated in patients who have a family member with psoriasis.

Psoriasis is an incurable, chronic, relapsing, systemic disorder associated with substantial health and economic burden (28, 29). An overall improvement in health outcomes is correlated with early detection and multidisciplinary approach tailored for individual patient aiming to provide comprehensive care and early identification of extracutaneous co-morbidities (28). Considering the findings of this study, we recommend routine screening for psoriasis in MS, particularly those with a family history of psoriasis. This has the potential to improve quality of life, cut costs and may also facilitate informed selection of MS DMTs. Future research should focus on large-scale, multicentre studies incorporating dermatologist-verified diagnoses to minimise diagnostic variability. Subgroup analyses, including comparisons of simplex and multiplex families, may further clarify genetic contributions and identify risk stratification in patients with concurrent MS and psoriasis.

## Limitations

Although we believe that the participants included in this study reflect the general Australian patients with MS to a reasonable degree, this study was underpowered (final sample size of 204 patients compared to target sample size was 379 participants), and the results of this study cannot be generalised across boarder populations. The target sample size was estimated based on MS prevalence in Australia [23,700 people] assuming a confidence interval of 95, 5% margin of error, and power of 0.8. Falling short of the target sample size reduces the precision of the estimates, increases the likelihood of type II error, and limits our ability to detect small but potentially meaningful associations. Consequently, some findings may not have reached statistical significance simply due to insufficient power rather than the absence of a true effect.

The observational nature of the study warrants cautious interpretation of its results. Given all participants in this study were recruited from a single MS clinic, sampling bias cannot be excluded. Moreover, reliance on self-reported data may introduce measurement error and information bias, particularly recall and reporting bias, which may influence the overall outcomes of interest. This is particularly relevant for MS patients with cognitive impairment, who may have been less likely to report psoriasis diagnoses in family members. Furthermore, while ethnicity is known to play a significant role in the epidemiology of MS and several autoimmune conditions (2, 30), ethnicity data were not collected within the scope of this study, so we were unable to evaluate the influence of ethnic differences on the observed outcomes.

Another limitation includes the validity of psoriasis diagnosis in this cohort, as some cases were confirmed by dermatologists while other were based on GP assessment which may lead to diagnostic variability.

## Conclusion

This study further supports the presence of a link between MS and familial psoriasis and provides valuable data for future genetic research. A larger-scale prospective study investigating mechanism of inheritance of psoriasis among MS patients is warranted to determine the characteristics of association in MS individuals with psoriasis or/and family history of psoriasis.

## Data Availability

The raw data supporting the conclusions of this article will be made available by the authors, without undue reservation.
